# Imaging methods to monitor and quantify cell differentiation

**DOI:** 10.3389/fcell.2025.1584858

**Published:** 2025-05-13

**Authors:** Kevin Cheah, Pingjin Chu, Greta Schmidt, Suzanne Scarlata

**Affiliations:** Department of Chemistry and Biochemistry, Worcester Polytechnic Institute, Worcester, MA, United States

**Keywords:** fluorescence imaging, neuronal cells, fibroblasts, differentiation, RNA imaging

## Abstract

The transition of a cell from a stem to a differentiated state involves an interrelated and complex series of events. These events include dynamic changes in cellular nucleic acid and protein content that are mediated by both intrinsic and extrinsic factors which ultimately lead to differentiation into specific lineage. Quantifying the parameters associated with differentiation and their changes under different conditions would not only allow for a better understanding of this process but also would enable the development of approaches that control differentiation. Here, we describe processes associated with the differentiation of two types of cultured cells, neurons and fibroblasts, and the tools to follow changes in real time. Specifically, we discuss methods to the identify cell lineage, changes in morphology, shifts in specific mRNA and miRNA levels as well as the changes in protein localization, interactions and assemblies that accompany differentiation.

## Overview

While most cells in the human body are fully differentiated, stem cells play a key role in the regeneration of skin, the gastrointestinal tract, muscle tissue and even neurons. The transition from a stem cell to a defined phenotype is a carefully orchestrated series of events that transform the physiology and characteristics of the cell (for background see (Hendriks et al., 2022; Sánchez Alvarado and Yamanaka, 2014; Newman, 2020; Nelson, 2022)). Differentiation involves dynamic changes in gene expression, protein synthesis, and cellular morphology driven by intrinsic genetic programs and extrinsic environmental cues. Even minor changes in these processes can result in cell dysfunction and/or irregular growth that could additionally impact the physiology of surrounding cells. Learning about the changes that occur during cell differentiation may enable the development of methods to control the differentiation process. While traditional methods to study differentiation involve cell disruption, recent tools allow us to follow changes in the levels and localization of proteins and nucleic acids in live cells during differentiation.

This review focuses on the tools and models used to study cell signaling and differentiation, with an emphasis on quantitative approaches that provide real-time insights into cellular processes. Specifically, it highlights imaging tools and molecular techniques that have been employed to track differentiation in various cells, including neuronal and fibroblast lines. These cell lines serve as model systems for studying diverse aspects of lineage selection and transitional outcomes.

## Neuronal differentiation models

There are several valuable cultured cell lines that are used to study neuronal differentiation, and these are chosen by the species origin, differentiation cues, and experimental goals. The most widely used model lines are rat PC12, human NT2 and SH-SY5Y and variant strains. PC12 cells are models for differentiation into sympathetic neurons (Greene and Tischler, 1976), while human NT2 cells are models for human neurodevelopment and neural stem cell differentiation (Pleasure and Lee, 1993) and SH-SY5Y cells are models for neurodegenerative diseases, neurotoxicity screening, and highthroughput drug testing, owing to their catecholaminergic properties, and adaptability (see Table 1) (Dravid et al., 2021; Xicoy et al., 2017).

**TABLE 1:** Comparison of PC12, NT2, and SH-SY5Y cells in neuronal differentiation (Greene and Tischler, 1976; Pleasure and Lee, 1993; Kovalevich and Langford, 2013; Musch et al., 2010).

Feature	PC12	NT2	SH-SY5Y
Origin	Rat pheochromocytoma	Human embryonal carcinoma (NTERA-2)	Human neuroblastoma
Differentiation Inducers	Nerve Growth Factor (NGF)	Retinoic Acid (RA) Cytosine-β-Darabinofuranoside (AraC)	Retinoic Acid (RA), BDNF, dbcAMP, phorbol esters
Differentiation Time	∼4 days	∼18 days	∼7–14 days
Neuronal Morphology	Neurite outgrowth, sympathetic neuron-like	Post-mitotic neurons, radial glial-like precursor phase	Neurite outgrowth, catecholaminergic neuron-like
Key Markers	βIII-tubulin, synapsin, TrkA	βIII-tubulin, MAP2, Nestin	βIII-tubulin, MAP2, synaptic proteins
Neurotransmitter Profile	Dopamine, norepinephrine, catecholamines	Glutamate and GABA	Dopamine, norepinephrine
Applications	Receptor-mediated signaling, neurite outgrowth, neurotoxicity	Neurodevelopment, neural stem cell studies, neurodegeneration	Neurotoxicity, neurodegeneration (esp. Parkinson’s disease), highthroughput screening

**FIGURE 1:**
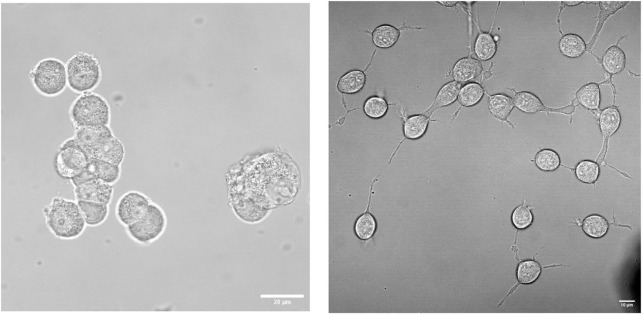
Morphological differences between undifferentiated and differentiated PC12 cells. Representative phase-contrast images showing undifferentiated PC12 cells (*left*) and PC12 cells treated with NGF after 48 h (*right*), from Schmidt and Scarlata, unpublished.

PC12 cells are not neuronal in origin but were derived from rat pheochromocytoma. In their undifferentiated state, PC12 cells display morphological and physiological traits resembling those of adrenal gland cells (Wiatrak et al., 2020). When treated with Nerve Growth Factor (NGF), they show pronounced changes in morphology with round or triangular flat somas and with ∼3 neurite extensions that overall resemble sympathetic ganglion neurons (see Figure 1). Along with these morphological changes, NGF treatment is accompanied by a loss in stem cell transcription factors and the appearance of synaptic vesicles. Compared to other neural models that typically require around 18 days to differentiate, PC12 cells differentiate in 2–3 days (Sierra-Fonseca et al., 2014; Greene and Tischler, 1976; Guroff, 1985). Biochemically, NGF binds to membrane-spanning tropomyosin receptor kinases A (TrkA) that mediate key signaling pathways allowing for neurite growth, cytoskeletal remodeling, and the secretion of neurotransmitters, including dopamine, norepinephrine, and other catecholamines (Kaplan et al., 1991). Differentiated PC12 cells also express ion and neurotransmitter receptors, further enhancing their relevance as a neural model system.

**FIGURE 2:**
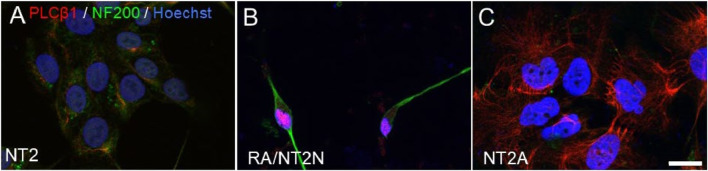
Changes in morphology of NT2 cells during differentiation. **(A)** Undifferentiated NT2 cells immunostained for phospholipase Cβ1 (*red*) showing low expression levels, the neuronal cell marker neurofilament 200 (*green*) which is not seen in undifferentiated cells and the nuclear stain Hoechst (*blue*). **(B, C)** show these same cells after 28 days of treatment with RA, where the cells take on a neuronal **(B)** or glial **(C)** morphology. Adapted from (González-Burguera et al., 2024).

NT2 cells, derived from the human embryonal carcinoma cell line NTERA2, are another common neuronal cell differentiation model (Pleasure and Lee, 1993). In their undifferentiated state, NT2 cells retain characteristics of embryonal carcinoma cells (Andrews, 1984). When treated with retinoic acid (RA), NT2 cells undergo stepwise differentiation over 4 weeks, leading to the formation of post-mitotic neurons (Pleasure and Lee, 1993) along with a smaller population of glial-like cells (Marchal-Victorion et al., 2003b; Musch et al., 2010) (see Figure 2). This process involves substantial changes in gene expression and cellular morphology that resemble early neural development. NT2-derived neurons display key neuronal characteristics, including neurite outgrowth, synapse formation, and neurofilament protein expression. A unique feature of NT2 cells is their resemblance to radial glial cells, which serve as neuronal progenitors, and provide structural support for neuronal migration (Marchal-Victorion et al., 2003a). This property makes NT2 cells an important model for studying early neurogenesis and neuronal lineage commitment.

Another cell line used to study neuronal cell differentiation is SH-SY5Y, a subclone of SKN-SH derived from human neuroblastoma (Shipley et al., 2016). Under standard culture conditions, SH-SY5Y cells exhibit both neuroblast-like and epithelial-like phenotypes (Kovalevich and Langford, 2013). SH-SY5Y cells can be differentiated into neuron-like cells using RA as well as other treatments. After RA differentiation, SH-SY5Y cells can be further matured using agents such as brain-derived neurotrophic factor (BDNF), dibutyryl cyclic AMP (dbcAMP), or phorbol esters (Kovalevich and Langford, 2013). These treatments enhance neuronal morphology, synapse formation, and neurotransmitter production, particularly dopamine and norepinephrine. Differentiated SH-SY5Y cells exhibit catecholaminergic neuronal characteristics, making them valuable models for Parkinson’s disease and other neurodegenerative disorder studies (Xicoy et al., 2017).

## Signaling mechanisms associated with neuronal differentiation

Neuronal differentiation allows neural progenitor cells to differentiate into specialized neurons and glial cells (Moazeny et al., 2022; Navarro Quiroz et al., 2018). Differentiation involves a complex series of interconnected and dynamic changes in protein-protein interactions (PPIs) that govern key physiological and pathological processes such as cell proliferation, differentiation, apoptosis, and signal transduction. The transition from pluripotency to differentiation is regulated by signaling molecules, transcription factors, and changes in PPIs that help guide lineage specification, axonogenesis, and synaptic maturation. Activation of TrkA receptors, binding of RA to retinoic acid receptors, or other initiation events induce a series of changes in PPIs that perpetrate signal transduction, transcriptional regulation, and cytoskeletal dynamics. Essential pathways like RA signaling, the MAPK/ERK cascade, and Rap1-mediated adhesion regulate gene expression programs governing neuronal fate. Transcription factors such as Oct4, POU5F1, NANOG, and PAX6 are key regulators of neural differentiation, and this process is marked by the downregulation of pluripotency markers (POU5F1, NANOG) and the upregulation of neurogenic factors (PAX6) (Moazeny et al., 2022). Bioinformatics-driven analyses of PPI networks have uncovered regulatory sub-networks essential for neural differentiation (Moazeny et al., 2022). Distinct interaction clusters regulate neuron differentiation, axon guidance, and retinol metabolism, highlighting the interconnected nature of neural commitment. Deciphering PPI networks advances our understanding of neurogenesis and may reveal therapeutic targets for neurodevelopmental and neurodegenerative disorders.

Neuronal differentiation is tightly regulated by multiple signaling pathways and dynamic protein relocalization. The MAPK/ERK, PI3K/AKT, Wnt/β-catenin, and Notch pathways are critical for initiating and sustaining differentiation through transcriptional and translational regulation. The MAPK/ERK pathway activates transcription factors like CREB, inducing neuronal gene expression (e.g., BDNF and c-fos) essential for synaptic plasticity and learning (Impey et al., 1998; Rhim et al., 2016; Sun et al., 2015). The PI3K/AKT pathway supports neuronal survival by preventing apoptosis and promoting protein synthesis that stabilize newly differentiated neurons (Brunet et al., 2001). Wnt/β-catenin signaling governs neural lineage commitment by promoting β-catenin nuclear translocation and activating transcription factors like NeuroD1, which drive neuronal fate determination (Kuwabara et al., 2009). Notch signaling maintains the balance between progenitor cells and differentiated neurons by suppressing neurogenic gene expression and preventing premature differentiation (Lasky and Wu, 2005).

**FIGURE 3:**
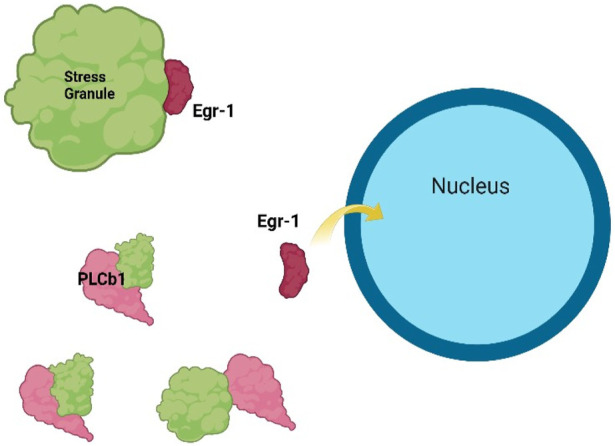
Example of how PC12 cell differentiation can be mediated by protein localization. In undifferentiated cells, the transcription factor Egr-1 is held in the cytosol through the association of stress granule proteins (*top left*), but increased levels of PLCβ1 dissolve stress granule complexes (*bottom left*) promoting the dissociation of Egr-1 and allowing relocalization to the nucleus to drive the transcription of genes associated with differentiation. (Qifti et al., 2021; Jackson et al., 2022a, Wei et al., 2015). Adapted from (González-Burguera et al., 2024) and drawn using BioRender.

All of these complex and interconnected PPIs depend on the local levels of the individual proteins that depend on their expression, turnover and protein localization that depends on the dynamic partitioning into cellular organelles or sequestration into membraneless inclusions. Accurate spatial and temporal protein distribution is crucial for intracellular signaling, transcriptional regulation, and cellular differentiation (Hamby et al., 2008), and protein mislocalization can cause cellular dysfunction, often leading to neurodevelopmental and neurodegenerative diseases (Chung et al., 2018). Alongside pathway activation, neuronal differentiation involves critical protein relocalization events that regulate gene expression at both the transcriptional and translational levels, such as the mechanism shown in Figure 3 *(see below).* Several key proteins undergo dynamic shifts between cellular compartments to modulate neuronal differentiation (Table 2).

**TABLE 2:** The role of protein localization in neuronal differentiation (Impey et al., 1998; Rhim et al., 2016; Sun et al., 2015; Kuwabara et al., 2009; González-Burguera et al., 2024).

Protein	Relocalization event	Functional outcome	Pathway involved
CREB	Translocates to the nucleus upon phosphorylation	Drives neuronal gene transcription, activating *BDNF* and *c-fos*	MAPK/ERK
β-Catenin	Accumulates in the nucleus in response to Wnt signaling	Promotes neural differentiation by activating *NeuroD1*	Wnt/β-catenin
Egr-1	Shifts between cytoplasm and nucleus based on differentiation cues	Modulates neuronal fate by competing with CREB for CBP binding	PLCβ1-mediated signaling

The dynamics of key signaling proteins and their spatial dynamics shape neuronal identity, support synaptic plasticity, learning, and memory formation. Consider the case of cAMP response element binding protein or CREB, which is a key transcription factor in neuronal differentiation and associated with learning and memory (Silva et al., 1998). CREB is activated by ERK phosphorylation, enabling it to bind CREB-binding protein (CBP) and initiate neurogenic gene transcription (Impey et al., 1998). Egr-1 competes with CREB for CBP binding, functioning as a regulatory switch that promotes or suppresses CREB-dependent transcription (Silverman et al., 1998). Disruptions in any of these pathways, such as abnormal β-catenin accumulation, Egr-1 or CREB mislocalization, are linked to neurological disorders, including schizophrenia, Alzheimer’s disease, and epileptic encephalopathy.

## An example of differentiation mediated by changes in protein localization

Neuronal signaling modulated by agents that can activate the Gαq/phospholipase Cβ (PLCβ)/phosphoinositide 4,5 bisphosphate (PIP2) pathway. This pathway responds to neurotransmitter such as acetylcholine and serotonin to generate calcium signals, and these signals are responsible for cytoskeletal changes, changes in the transcription CREB and other proteins associated with learning and memory, as well as the release of synaptic vesicles (Rennie et al., 2022). Even though this pathway is distinct from the NGF/TrkA and other growth factor pathways, our lab has found that down-regulating PLCβ prevents NGF-induced differentiation of PC12 cells (Garwain et al., 2018). However, differentiation still occurs when Gαq is downregulated suggesting that PLCβ works through a pathway independent of its normal calcium-mediating function. Down-regulating PLCβ in fully differentiated PC12 or SK-N-SH cells returns them to the undifferentiated state (Garwain et al., 2020). Over-expressing PLCβ in undifferentiation cultured or primary cells induces differentiation without the need for NGF, retinoic acid or other differentiation factors (González-Burguera et al., 2024).

To understand the mechanism through which PLCβ controls differentiation, we noted that the onset of differentiation is associated with a large increase in PLCβ expression that localizes in the cytosol (Garwain et al., 2020; González-Burguera et al., 2024). This cytosolic population was found to bind to proteins involved in RNA-induced silencing and the formation of stress granules. It also modulates the localization of the transcription factor, Early Growth Response 1 (Egr-1). At low levels of PLCβ, Egr-1 localizes in the cytosol where it binds to stress granule - associated proteins that stabilize this localization. However, high cytosolic PLCβ levels dissolve these stress granules releasing Egr-1, which then transits to the nucleus where it promotes the transcription of genes associated with the differentiated state (González-Burguera et al., 2024) (Figure 3).

## Fibroblast differentiation

The term ‘fibroblast’ generically refers to a type of connective tissue cell that can produce collagen. It was first described as distinct spindle-shaped cells embedded in fibrous connective tissue (Virchow, 1858). Fibroblasts are ubiquitous in various tissues throughout the body. Under normal conditions, they are in a quiescent state and contribute to the production and maintenance of the extracellular matrix (ECM) which is a complex network of molecules that provide structural and biochemical support to surrounding cells. In response to tissue repair processes, fibroblasts undergo a phenotypic change into a contractile state, leading to increased ECM production and contraction. During tissue repair or injury, fibroblasts differentiate into myofibroblasts, which also plays a crucial role in pathological settings, particularly pathological fibrosis, tissue scarring in organs and stromal response around tumors (Darby and Hewitson, 2007).

**FIGURE 4:**
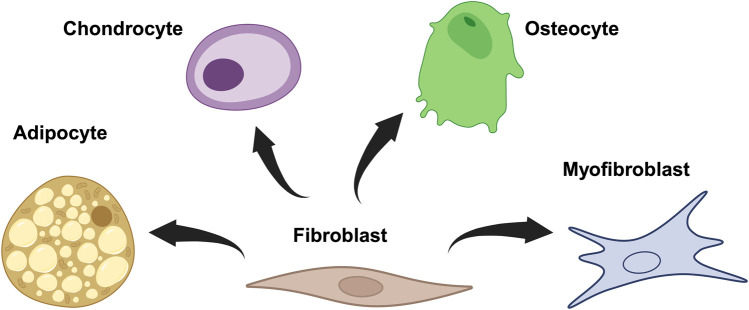
Fibroblasts can be induced to differentiate into adipocyte, chondrocyte, osteocyte, and myofibroblast. Created in https://BioRender.com.

Actin is the most abundantly expressed protein in most eukaryotic cells. While β- and γ-actins are ubiquitously expressed, the α-cardiac, α-skeletal, and α-smooth muscle isoforms exhibit tissue-specific expression. The expression of α-smooth muscle actin (α-SMA) is commonly used as a differentiation marker for myofibroblast. Mesenchymal stem cells (MSC) are well known for their ability to differentiate into various cell types, including bone, cartilage, and fat. However, MSCs have certain drawbacks, such as the required invasive harvesting procedures and limited *in vitro* expansion capacity due to changes in cellular phenotype. To address these challenges, Dastagir and colleagues demonstrated that NIH-3T3 and murine embryonic fibroblasts (MEFs) can be induced to differentiate into adipogenic, chondrogenic, and osteogenic phenotypes. These murine fibroblast cell lines serve as new models for investigating fibroblast differentiation processes (see Figure 4 and Table 3 for summary of various induced phenotypes of fibroblast) (Dastagir et al., 2014).

**TABLE 3:** Various induced phenotypes of fibroblast (Negmadjanov et al., 2015; Dastagir et al., 2014).

Phenotypes	Myofibroblast	Adipocyte	Chondrocyte	Osteocyte
Cell Line Used	NIH-3T3	NIH-3T3, MEF	NIH-3T3, MEF	NIH-3T3, MEF
Induced Differentiation Conditions	DMEM supplemented with 2.5% FBS and 5 ng/mL TGF-β1	DMEM F12 supplemented with 10% FBS, 393 ng/mL dexamethasone, 50 μg/mL ascorbic acid-2-phosphate 10 μL/mL Penicillin/streptomycin, 111.1 μg/mL 3-isobutyl-1-methylxanthine, 1 μg/mL insulin, and 35.8 μg/mL indomethacin	DMEM F12 supplemented with 39.3 ng/mL dexamethasone, 50 μg/mL ascorbic acid-2-phosphate, 10 μL/mL penicillin/streptomycin, 10 μL/mL insulin-transferrin-selenium, 22.5 μL/mL sodium pyruvate, 40 μL/mL proline, and 1 μL/mL TGF-β1	DMEM F12 supplemented with 10% FBS, 39.3 ng/mL dexamethasone, 50 μg/mL ascorbic acid-2-phosphate, 10 μL/mL penicillin/streptomycin, and 756 μg/mL β-glycerol
Differentiation Time	2 days	2 weeks	3 weeks	4 weeks
Differentiation Markers	α-SMA	Resistin, adiponectin	Elastin, aggrecan	Bone alkaline phosphatase (BALP) and RUNX2

## Signaling mechanisms associated with fibroblast differentiation

**FIGURE 5:**
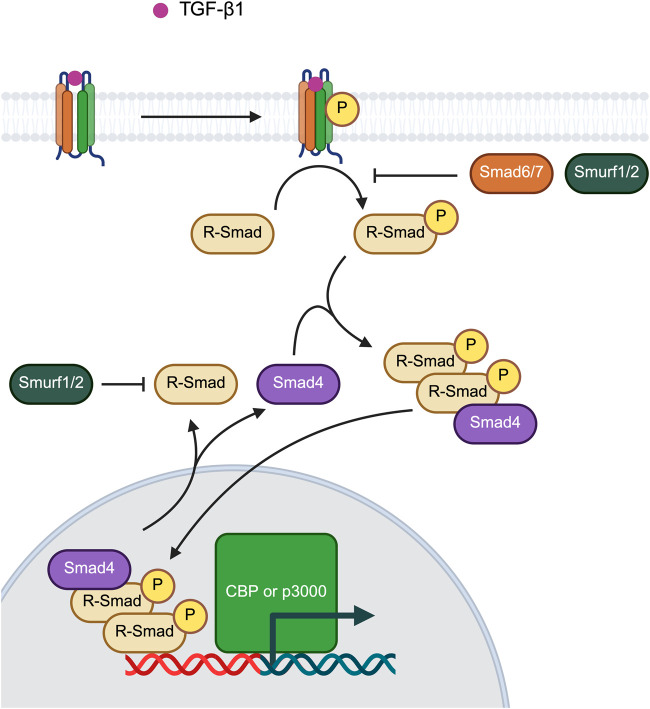
Canonical TGF-β signaling via Smad signaling pathway. Created in https://BioRender.com.

Transforming growth factor-β1 (TGF-β1) is a widely studied cytokine known for its role in inducing fibroblast differentiation. Canonical TGF-β signaling is mediated through the Smad signaling pathway (Figure 5). TGF-β receptors belong to a class of intrinsic serine/threonine kinases. Upon TGF-β1 binding, these receptors undergo autophosphorylation and subsequently phosphorylate receptor-regulated Smad (R-Smad). Two phosphorylated R-Smad subunits then associate with Smad4 to form a heterotrimeric complex. This complex translocates to the nucleus, where it interacts with transcription factors such as CBP or p3000 to regulate gene expression. Apart from Smad signaling pathway, non-canonical TGF-β signal transduction occurs through Smad independent pathways including JAK/STAT, MAPK–ERK, RAF–MEK1/2–ERK1/2, p38 MAPK, Jun kinase, NF-κB, PI3K–AKT–mTOR, and Rho–associated kinase (ROCK) (Derynck and Budi, 2019; Zhang, 2009).

## Mechanisms of myofibroblast mechanotransduction

Experimental models investigating tissue injury and fibrosis often involve studying myofibroblast activation through mechanical stress (Lagares and Hinz, 2021). Among the various mechanical factors studied, the stress exerted by the ‘stiffness’ of wound and fibrotic ECM represents a critical mechanism driving myofibroblast activation. The shift in ECM stiffness following tissue damage serves as a fundamental signaling mechanism, enabling fibroblasts to modulate their functional state and activity in response to the tissue repair. Through different cell surface receptors such as integrins, discoidin domain receptors (DDR), G protein-coupled receptors (GPCR) and stretch-activated ion channels (SACs), fibroblasts can probe and respond to mechanical cues transmitted from the ECM (Saraswathibhatla et al., 2023). These receptor systems constitute the mechanotransduction signaling pathways that regulate fibroblast activation.

In response to tissue injury, fibroblasts relay forces to contract the ECM into scar tissue. By physically linking their contractile cytoskeleton to transmembrane ECM adhesion receptor complexes, fibroblasts can probe the chemical and mechanical properties of their environment (Kanchanawong and Calderwood, 2023). Some widely studied fibroblast ECM receptors include the hyaluronic acid receptor CD44, DDR1 and DDR2, and various integrins. Hyaluronic acid binding to CD44 receptor leads to formation of adhesion complexes composed of adaptor proteins which are linked to the contractile actin cytoskeleton (Govindaraju et al., 2019). CD44 activation and its downstream effectors are essential for fibroblast motility and invasion in response to ECM stiffening. Deletion of CD44 has been shown to impair the stiff remodeling of primary murine dermal fibroblasts (Razinia et al., 2017).

DDR1 has garnered considerable research interest due to its specific binding and compaction of fibrillar collagen through a mechanically regulated process driven by myosin (Coelho et al., 2017). Overexpression of DDR1 on fibroblasts has been observed in tissues affected by fibroproliferative disorders such as skin hypertrophic scars, cancer, liver, kidney and lung fibrosis (Moll et al., 2019; Guerrot et al., 2011).

The binding of integrin β1 to various α-integrins results in the formation of heterodimers α1β1, α2β1, α10β1, and α11β1. These fibroblast collagen receptors are implicated in tissue repair, including skin and heart (Sawant et al., 2021; Li and Frangogiannis, 2022). Additionally, Schulz and coworkers reported reduced fibroblast activation and skin scarring in an α11β1-knockout model (Schulz et al., 2015). Integrin α1β8 can bind multiple ECM ligands through its recognition of the arginine–glycine–aspartate (RGD) motif. This motif can be found in multiple ECM proteins including vitronectin and fibronectin. These proteins play important roles in formation of provisional ECM after injury (Frangogiannis, 2017; Klingberg et al., 2013). Similarly, the latent TGFβ1–latency-associated peptide (LAP) complex contains an RGD motif in its key recognition site. Integrin αvβ1, predominantly found in fibroblasts, binds to TGFβ1–LAP with high-affinity through the RGD motif. This binding activates latent TGFβ1, as observed in the extracellular space of *in vitro* and *in vivo* fibrosis models (Lodyga and Hinz, 2020; Massagué and Sheppard, 2023).

During the maturation of integrin adhesions, talin and kindlin family members bind directly to the integrins tail and the actin cytoskeleton (Goult et al., 2018; Sun et al., 2019). Studies have shown that kindlin-2 is upregulated in experimental models of fibrosis in the heart, kidney, lung, and skin. Functional ablation of kindlin-2 prevents mechanical fibroblast activation (Godbout et al., 2020; Zhang et al., 2021c). Other crucial mechanosensitive elements within fibroblast adhesions include focal adhesion kinase (FAK) and integrin-linked kinase (ILK). Functional loss of FAK in cultured fibroblasts decreases myofibroblast activation (Thannickal et al., 2003). Similarly, ILK plays a role in fibroblast force transduction, TGFβ1 secretion, myofibroblast activation, and myofibroblast-mediated tissue contraction in murine dermal and renal fibrosis (Yamamura et al., 2023; Nüchel et al., 2018).

In fibroblasts, latent TGFβ1 is secreted in complex with its pro-peptide LAP. Prior to secretion, LAP is intracellularly cleaved by furin enzymes. The noncovalent interaction between cleaved LAP and TGFβ1 helps retain TGFβ1 in an inactive ‘straitjacket’ configuration. The longterm storage of latent TGFβ1 in the ECM is facilitated by the covalent binding of LAP to the fibrillin protein family member, latent TGFβ1-binding protein 1 (LTBP1). In myofibroblasts, LTBP1 is organized into straighter and denser fibrils compared to fibroblasts. The ECM must be sufficiently stiff to induce a conformational change in TGFβ1–LAP, overcoming the cell forces required to liberate TGFβ1 from its LAP straitjacket configuration (Klingberg et al., 2014). These findings suggest that the first mechanical checkpoint for myofibroblast activation is the mechanical activation of TGFβ1.

SACs are localized to the fibroblast membrane and can be activated by mechanical strain in response to extracellular stress, leading to coordinated myofibroblast activation and activity (Stewart and Turner, 2021; Adapala et al., 2021). The SAC transient receptor potential vanilloid type 4 (TRPV4) can interact with both integrin β1 and DDR1 adhesions. This interaction coordinates fibroblast ECM remodeling by regulating the local influx of Ca^2+^ ions (Wang et al., 2022a; Matthews et al., 2010). Functional ablation of TRPV4 prevents experimentally induced fibrosis in the mouse heart, suggesting that TRPV4 is essential for myofibroblast activation in skin, lung, and cardiac fibroblasts (Grove et al., 2019; Zou et al., 2022; Sharma et al., 2017). Similarly, TRVP3 has been shown to regulate myofibroblast activation of dermal fibroblasts via the Smad2 and Smad3 pathways (Um et al., 2020). Following the discovery of Piezo1 and Piezo2 as another class of SACs (Coste et al., 2010), Piezo1 was revealed to play a crucial role in regulating fibroblast repair functions. Upon mechanical stimulation, Piezo1 elicits a signaling cascade that leads to myofibroblast activation. Much like TRPV4, Piezo1 localizes in adhesions, where it plays an important role in probing fibroblast ECM stiffness (Yao et al., 2022).

Myofibroblasts contraction is primarily regulated by myosin light chain (MLC) kinase (MLCK)-mediated phosphorylation of the MLCs associated with non-muscle myosin 2 (MYH2). At least two modes of contraction are observed in myofibroblasts: one regulated by stimulating MLCK activity via Ca^2+^ signaling and the other by inhibiting MLCP activity via RhoA–ROCK signaling (Castella et al., 2010; Parizi et al., 2000). Through RhoA–ROCK, ligand binding to GPCR lysophosphatidic acid (LPA) receptor 1 enhances contraction and contribute to myofibroblast activation (Sakai et al., 2017; Ungefroren et al., 2018; Zhang et al., 2022). In addition to ROCK-mediated contraction, GPCR signaling regulates myofibroblast activation at the level of transcription by interacting with other pathways, such as JAK2–STAT3 (Oh et al., 2018), nuclear factor of activated T cells (NFAT) (Eguchi et al., 2021), and YAP/TAZ signaling (Haak et al., 2019).

Yes-associated protein (YAP) and transcriptional adaptor putative zinc finger (TAZ) were first discovered as components of Hippo pathway. Various upstream signals regulate YAP/TAZ, including those activated by ECM, mechanical stress, mitogens, cell polarity, cell adhesions, GPCRs, tyrosine kinase receptors, and changes in cellular metabolism (Meng et al., 2016). At its core, Hippo pathway is a mammalian kinase cascade composed of Ste20-like kinase 1 (MST1; also known as serine–threonine-protein kinase 4) and MST2 (also known as STK3), large tumor suppressor kinase 1 (LATS1) and LATS2, the adaptor proteins Salvador 1 (SAV1), MOB1A and MOB1B, the homologous transcriptional co-activators YAP and TAZ, and the TEAD transcription factors. YAP/TAZ binds to TEAD, which then interacts with chromatin-remodeling factors and modulates RNA polymerase II (Pol II), ultimately regulating gene expression (Moya and Halder, 2019). Beyond their canonical role in Hippo signaling, YAP and TAZ also function as mechanosensitive transcription factors. A shift in the cytosolic population of YAP/TAZ in response to ECM stiffness has been reported (Dupont et al., 2011). Following activation of RhoA, the formation of stress fibers inhibits LATS1 and LATS2 in the cytoplasm, preventing the phosphorylation of YAP/TAZ, which would otherwise inhibit their transcription co-activator function. As a result, the cytosolic population of unphosphorylated YAP/TAZ increases, allowing them to translocate into the nucleus and regulate gene expression by associating with TEAD, Smads, p73 and RUNX1. In a study investigating YAP/TAZ signaling inhibition, experimentally induced fibrosis was reduced (Szeto et al., 2016). Furthermore, TAZ transcription has been shown to depend on another mechanosensitive co-transcription factor—myocardin-related transcription factor A (MRTFA) In response to mechanical stress, MRFTA is initially sequestered by binding to non-polymerized globular actin, leading to a shift in its cytosolic population. Following stress-induced actin polymerization in the cytosol, MRFTA is released from sequestration, allowing it to translocate to nucleus and act as a co-transcription factor alongside with serum response factor (SRF) to regulate gene expression (Miranda et al., 2021). Some examples of MRTF–SRF target genes are myofibroblast-associated genes *CCN2* and *ACTA2,* as well as genes encoding stress-sensing and stress-transmitting cytoskeletal components, such as integrins α2, α11, αv, β1, β3, β5, ILK and TAZ (Yamamura et al., 2023).

## General strategies to follow differentiation

These descriptions of neuronal and fibroblast differentiation pathways illustrate the many environmental cues that can induce differentiation. These specific external and internal conditions can manifest into specific cell lineages. Given the complexity of differentiation and the many possible molecular interactions that give rise to different lineages, the study of differentiation may appear unachievable. Here, we describe methods to follow differentiation in cells as they occur. We acknowledge that almost all of these methods involve some knowledge of the molecules involved generating some bias, as discussed below, but will give valuable information that can be incorporated into systems analysis and other tools to generate predictive models for differentiation. Incorporating these approaches with unbiased methods such as RNAseq and proteomics can help identify the best molecules and pathways to study, and ultimately, help delineate potential mechanisms to investigate.

What are the best ways to follow differentiation in real time? While the factors that generate differentiation ultimately occur on the protein level, we thought it would be easiest to first describe methods to follow changes in the nucleic acids that give rise to the differentiated state, followed by the changes in cell morphology and protein associations that accompany these changes. As can be seen, all of these involve optical microscopy.

## Following differentiation by live-cell RNA imaging

**FIGURE 6:**
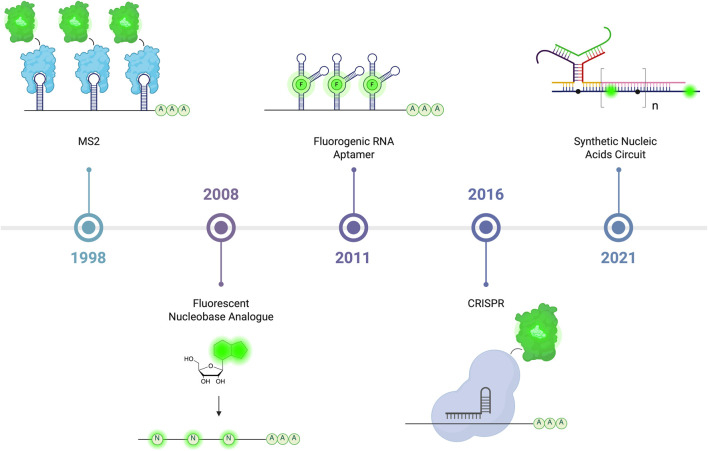
The development of live-cell RNA imaging starting with a system relying on the ability of stem-loop to recruit RNA bacteriophage MS2 coat protein (MCP) developed in 1998. Following this are methods where fluorescent nucleobase analogues can be *in vitro* transcribed or metabolically incorporated to form fluorescent transcripts, and after are fluorogenic RNA aptamers that rely on binding small-molecule fluorophore to emit light. Also depicted are methods that involve the conjugation of fluorescent protein to RNA-binding Cas protein that allow endogenous RNA visualization, and followed by synthetic nucleic acids probes with recognition domains that upon binding to target transcript, assemble into fluorescence-emitting probe. Created in https://BioRender.com.

In mammalian cells, differentiated cell types execute a wide array of specialized functions that rely on genes selectively expressed in that cell type. A typical differentiated cell expresses approximately half the genes in its genome. This selective gene expression, which varies between cell type, can be investigated by studying the transcriptome and proteome. After transcription, RNA undergoes modifications such as splicing, localization, translation and degradation. These processes are highly regulated both spatially and temporally. Therefore, expanding the toolbox used to visualize RNA is essential to understanding how RNA localization and dynamics influence function. Here, we review tools developed to tag and track RNA in living cells, with an emphasis to those applied to live mammalian cells RNA imaging (see Table 4 and Figure 6).

**TABLE 4:** Summary of RNA imaging tools.

RNA imaging tools	RNA imaged	References
Fluorescent nucleobase analogue (See Figure 7)	mRNA	Baladi et al. (2021), Lang et al. (2023), Nilsson et al. (2023), Steinbuch et al. (2024), Wang et al. (2022c)
Fluorogenic RNA aptamer	mRNA, miRNA	Bühler et al. (2023), Dou et al. (2021), Dou et al. (2022), Englert et al. (2023), Fam et al. (2022), Gu et al. (2021), Jiang et al. (2023b), Peng et al. (2024), Peng et al. (2023), Sarfraz et al. (2023), Sunbul et al. (2021), Wang et al., (2022d), Zhang et al. (2021b), Zheng et al. (2024a), Zheng et al. (2024b), Zhou et al. (2021), Zuo et al. (2024), Peng et al. (2022)
CRISPR	mRNA	Chen et al., (2022a), Jia et al. (2024), Tang et al. (2023), Tang et al. (2022), Wang et al. (2022b)
RNA-binding protein	mRNA	Eguchi et al. (2023), Halbers et al. (2024)
Synthetic nucleic acid circuits (see Figure 8)	mRNA, miRNA	Chen et al. (2022b), He et al., (2022), Huang et al., (2023), Jiang et al. (2024), Mo et al. (2024a), Mo et al. (2024b), Wang et al. (2023a), Xu et al., 2024, Xu et al., 2022, Zhao et al., 2022, Dai et al., 2024, Wan et al. 2021)
Other methods	mRNA, miRNA	Jiang et al., 2023a, Wang et al., 2023b, Yadavalli et al., 2024, Zhang et al., 2021a, Zhu et al. (2023)

### RNA-binding protein (RBP)

RNA-binding proteins can bind to specific RNA sequences through protein-RNA interactions. For example, the RNA bacteriophage MS2 coat protein (MCP) can bind to a specific RNA stem-loop. Together with other RBP such as PP7 bacteriophage coat protein (PCP) and pumilio homology domain of human pumilio 1 (PUM-HD), RBP continues to emerge as popular RNA labeling scheme. In 2023, Eguchi and colleagues developed a bioluminescence-based RNA probe that incorporates a split NanoLuc reporter with two PUM-HD mutants RNA-recognition domain (Eguchi et al., 2023). More recently in 2024, Halbers and colleagues developed a reporter that features split NanoLuc fused to MCP and PCP. It was reported only a single copy of RNA stem-loop is required to achieve high sensitivity (Halbers et al., 2024).

### Fluorescent nucleobase analogue (FBA)

**FIGURE 7:**
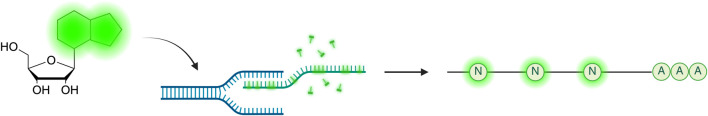
Diagram showing how fluorescent nucleobase analogue (FBA) can be *in vitro* transcribed or metabolically incorporated into RNA leading to formation of fluorescent transcripts. Created in https://BioRender.com.

RNA imaging tools generally require extensive modifications, which can potentially alter their intrinsic properties. Therefore, it is essential to develop a robust RNA labeling scheme that minimally perturbs RNA biology while remaining compatible with live-cell fluorescence imaging. FBAs are popular candidates for RNA labeling since they introduce relatively small chemical modification to the natural nucleobase they replace (Figure 7). Their optimized design preserves canonical Watson-Crick base-pairing and base-stacking of target nucleic acids. In 2021, Baladi and colleagues developed a fluorescent tricyclic cytosine analogue, tC^O^, which can replace up to 100% of the natural cytosine (Baladi et al., 2021). It was demonstrated that tC^O^-labelled mRNA is efficiently translated into functional protein whose fluorescence can be visualized using confocal microscopy. Similarly, Wang and colleagues showed that another class of fluorescent bicyclic and tricyclic cytidine analogues can be metabolically incorporated into RNA via the overexpression of uridine-cytidine kinase 2 (Wang et al., 2022c). Other FBAs, including adenine and guanine analogues, have also emerged as promising candidates for RNA live-cell imaging (Lang et al., 2023; Steinbuch et al., 2024). However, FBAs typically require UV excitation, which has drawbacks such as phototoxicity, poor tissue penetration, and photobleaching. In recent work by Nilsson and colleagues, a quadracyclic adenine analogue was shown to exhibit two-photon absorption, making it a viable option for RNA live-cell imaging (Nilsson et al., 2023).

### Fluorogenic RNA aptamer

In 2011, Paige and colleagues engineered an RNA aptamer capable of binding fluorophores (Paige et al., 2011). The fluorophore was designed based on the fluorophore formation in GFP. This RNA-fluorophore system, termed Spinach, garnered significant attention for its potential in developing RNA-fluorophore with improved photostability and modular excitation wavelength. In 2021, Sunbul and colleagues developed a new class of RNA aptamer, RhoBAST which binds rhodamine (Sunbul et al., 2021). RhoBAST was reported to bind rhodamine with rapid association and dissociation kinetics, exhibits diminished photobleaching, and high RNA-labeling signal-to-noise ratio. Several improvements have also been made to the RhoBAST system including the development of dimeric biRhoBAST (Bühler et al., 2023), and a spirocyclic rhodamine-based dye (Englert et al., 2023). In 2022, Fam and colleagues introduced o-Gemini, a dimeric self-quenching rhodamine dye that binds o-Coral tagged RNA, enabling high signal-to-noise RNA live-cell imaging (Fam et al., 2022). Additionally, considerable efforts have been made to address background fluorescence commonly observed in RNA labeling schemes. These include: a self-assembling Corn aptamer fragment for miRNA imaging (Gu et al., 2021), a degron-binding RNA aptamer that restores its active conformation upon target RNA binding, preventing the degradation of the fluorescent protein reporter (Zhou et al., 2021), an RNA aptamer that restores its fluorescence upon RNA-inducing silencing complex cleavage (Dou et al., 2022), an RNA aptamer that restores its active conformation in the presence of target RNA (Peng et al., 2023; Zheng et al., 2024a; Wang et al., 2022d), and a split RNA and split fluorescent protein system (Peng et al., 2024).

Live-cell RNA imaging is typically restricted to single gene per color, prompting the development of various strategies to overcome this limitation. Through systematic evolution of ligands by exponential enrichment (SELEX), researchers developed new aptamers such as Clivia (Jiang et al., 2023b) and Okra (Zuo et al., 2024). It was demonstrated that a single-excitation two emission dual-color imaging of RNA could be achieved by combining Clivia or Okra with Pepper. More recently, Zheng and colleagues demonstrated that multiplexing is possible through genetically incorporating multiple orthogonal aptamers into living cells (Zheng et al., 2024b). In 2021, Zhang and colleagues developed a near-infrared (NIR)-fluorescence RNA aptamer fluorophore complex to address challenges associated with single-color fluorescence activation (Zhang et al., 2021b). Additionally, Peng and colleagues engineered an organelle-targeting RNA aptamer, which selectively localizes in mitochondria and nucleus, enabling organelle-specific imaging (Peng et al., 2022). In 2021, Dou and colleagues developed a dual color RNA aptamer for ratiometric miRNA imaging. The RNA aptamer consists of SRB2 as the sensor module with Mango as internal reference (Dou et al., 2021).

The fluorogenic RNA aptamers described above typically use confocal microscopy to characterize their presence, localization and other properties. Alternately, in 2023, Sarfraz and colleagues demonstrated that fluorescence lifetime imaging microscopy (FLIM) can be employed to detect fluorescence using Riboglow platform, reporting improved cell contrast compared to intensity-based detection (Sarfraz et al., 2023).

### CRISPR

The discovery of CRISPR has led to advances in RNA live-cell imaging. In 2022, Mao and colleagues developed a CRISPR/dCas13a system incorporating the SunTag system, which recruits the split reporter Venus fluorescent protein (Chen et al., 2022a). Tang and colleagues demonstrated that Broccoli and Pepper aptamers can be embedded in the sgRNA sequence of the CRISPR/dCas13b system to label endogenous RNA (Tang et al., 2022). The delivery of the CRISPR/Cas system into living cells is often achieved through transfection. However, in 2022, Wang and colleagues reported that the CRISPR/Cas12a system can be delivered into living cells using MnO_2_ nanosheets to detect mRNA, outperforming commercial liposome carriers (Wang et al., 2022b). In 2023, Tang and colleagues reported that dCas13 can be tagged with Tat peptides, which recruit an RNA aptamer with a trans-activation response (TAR) attached to it. This approach enables RNA live-cell imaging with improved fluorescence brightness and signal-to-noise ratio (Tang et al., 2023). More recently, Jia and colleagues developed a CRISPR/dCas12a system capable of multiplexed RNA live-cell imaging. This strategy relies on Cas12a′s ability to process a pre-crRNA into mature crRNAs designed to target different RNA of interest (ROIs). The dCas12a was tagged with different fluorescent proteins, enabling multiplexed imaging (Jia et al., 2024).

### Synthetic nucleic acids circuit

**FIGURE 8:**
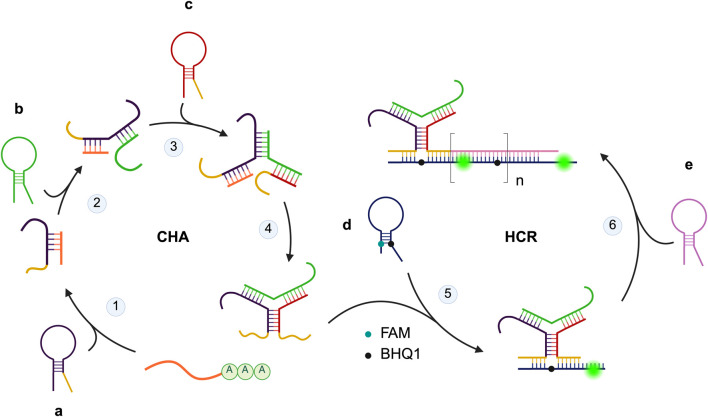
Example of synthetic nucleic acid circuit mechanism. The circuit consists of 5 hairpin species a–e, with a fluorophore-quencher pair labelled d. In the presence of target mRNA, a, b, and c assemble into the Y-shaped probe with sequence complementary to d via catalytic hairpin assembly (CHA). Hybridization of the probe with d leads to fluorescence turn-on. Signal amplification is achieved through hybridization chain reaction (HCR) where e hybridizes with d forming a “polymer” with multiple fluorescence turn-on. Created in https://BioRender.com.

Recently, there has been growing interest in developing RNA-labeling tools based on synthetic nucleic acids circuit as described in Figure 8. One of the key advantages of these tools is their ability to detect low-abundance RNA through *in situ* signal amplification. In 2021, Xu and colleagues developed an mRNA-sensing circuit based on toeholdmediated Toehold-mediated strand displacement (Xu et al., 2022). However, circuits relying on toehold-mediated strand displacement often suffer from sluggish reaction rates. To address this limitation, Mo and colleagues demonstrated that the toehold exchange of duplexes and hairpins polymers proceed faster than conventional toehold-mediated strand displacement of hairpins (Mo et al., 2024a). Catalytic hairpin assembly (CHA) and hybridization chain reaction (HCR) remain two of the most commonly operating mechanisms in synthetic nucleic acid circuits. For example, CHA circuits utilizing sensing strand functionalized on gold nanoparticles (NPs) (Huang et al., 2023) and polydopamine NPs (Xu et al., 2024) have been developed as platforms for mRNA imaging. Additionally, CHA circuits have been explored as tools for miRNA imaging. In 2022, He and colleagues developed a Y-shaped probe that assembles via CHA in the presence of endogenous mRNA (He et al., 2022). A similar approach was adopted by Mo and colleagues in 2024 (Mo et al., 2024b). In this work, hairpin polymers were integrated onto DNA tetrahedral nanostructure that assembles into the Y-shaped probe. The nanostructure was reported to accelerate the reaction rate and improve the HCR amplification efficiency. To further enhance the Y-shaped probe, 2 Mg^2+^-dependent E6 type RNA-cleaving DNAzymes I and II were incorporated. Wan and colleagues reported that signal amplification was achieved through the regeneration of the species that initiate the assembly of the Y-shaped probe and the cleavage of a fluorophore-quencher pair by the DNAzymes (Wan et al., 2021). Tetrazine ligation, a well-known bioorthogonal reaction, was first developed in 2008 (Blackman et al., 2008; Devaraj et al., 2008). It involves the cycloaddition of tetrazine with a dienophile, forming a cyclic adduct facilitated by extrusion of dinitrogen. In 2022, Zhao and colleagues developed a CHA circuit that relies on proximity-induced fluorophore formation of tetrazine with vinyl ether-caged pro-fluorophore for mRNA imaging (Zhao et al., 2022). A similar approach was applied in an HCR circuit developed by Chen and colleagues, where a black phosphorus-based carrier was used to deliver the system. The authors reported a high loading capacity and excellent biocompatibility (Chen et al., 2022b). APE1, an enzyme involved in DNA base excision repair, is also abundantly expressed in mitochondria. Dai and colleagues engineered a hairpin with APE1 cleavage site that can initiate cascading HCR upon cleavage, enabling mitochondrial miRNA imaging (Dai et al., 2024). Several circuits combining both CHA and HCR have also been reported. In one such circuit developed by Wang and colleagues, a linear hairpin polymer is formed through HCR upon binding to target miRNA. Signal amplification is then achieved through CHA in the presence of an endogenous mRNA catalyst (Wang et al., 2023a). Signal leakage often arise due to the constitutively activated mode of sensing probe. To address this limitation, Jiang and colleagues developed a self-localized cascade circuit. In the presence of mRNA, a stable dsDNA duplex is formed, and only in the presence of target miRNA, the HCR can proceed (Jiang et al., 2024).

### Other RNA visualization methods

Additional tools have been reported, including Ag nanoclusters for miRNA imaging (Yadavalli et al., 2024), polyA-mediated dual-color sticky flares for simultaneous imaging of two mRNA (Zhu et al., 2023), activatable fluorescence-encoded nanoprobe for multiplexing (Wang et al., 2023b), RNA-targeting red-emissive carbon dots (Jiang et al., 2023a), and organic dark quencher-encapsulated surface-cross-linked micelles (Zhang et al., 2021a).

## Methods for studying protein-protein interactions

Biochemical methods such as immunoprecipitation followed by Western blotting can be used to study protein-protein interactions and other markers of cellular differentiation, and while these methods are specific and sensitive, they cannot be performed on living systems. Biophysical methods include several fluorescence-based techniques that can be performed in live cells and resolve atomic structure of proteins and kinetics of protein interactions (Sarkar et al., 2024).

Fluorescence correlation spectroscopy (FCS) can be a useful tool for studying markers of cellular differentiation (Kim et al., 2007). In FCS, a laser excites a fluorescent sample, and the detected signal fluctuations provide information on kinetic properties of molecules at equilibrium. FCS requires a small observation volume, 10^−1^ μm which can be helpful when characterizing difficult to isolate samples. FCS samples must be fluorescent for proper interpretation, and instrument calibration must be done using a sample with a known diffusion coefficient. The autocorrelation function is an often-used statistical method to measure how long signal fluctuations persist and determine the diffusion coefficient and the average number of particles. Concentration of can also be calculated with the relative fluctuation amplitude of the signal (Müller et al., 2003).

**FIGURE 9:**
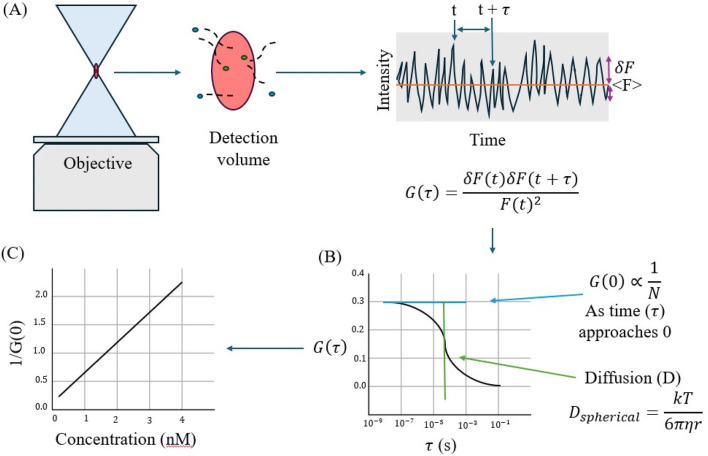
**(A)** Fluorescence correlation spectroscopy (FCS) follows signal intensity over time to determine the autocorrelation function G(T). **(B)** Autocorrelation function plotted against time can be used to determine the diffusion coefficient, which can then be used to determine the Stokes radius of the molecule (r). **(C)** The reciprocal of G (0) is proportional to the average number of molecules in solution (<N>) and can be plotted against concentration to determine N. Figure adapted from (Jameson, 2014).

In live cells, FCS has been used to study protein-protein interactions, protein-nucleic acid interactions, and protein organization on the plasma membrane (see Figure 9). Previously, our lab studied the diffusion of G-protein coupled receptors in solution using FCS and fluorescence recovery after photobleaching (FRAP) (Calizo and Scarlata, 2013). The small volume of FCS created a discrepancy between the two methods and suggested that confined diffusion was occurring. Our lab also used FCS to study the diffusion and concentration of amyloid precursor protein in stress granules (Jackson et al., 2022b). For more information on the use of FCS in live cells, see (Smith, 2024).

**FIGURE 10:**
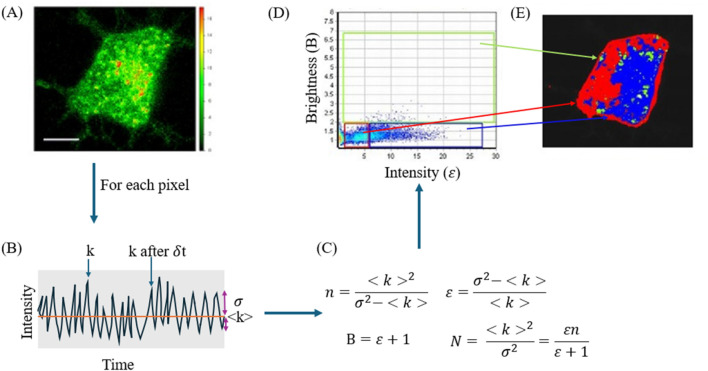
N&B analysis uses repeated scans of an XY image to determine the number of particles in solution and their aggregation state. **(A)** Image of a PC12 cell expressing a GFP tagged protein. **(B)** Simulated intensity versus time map showing the intensity at one point (k), the average intensity <k>, and the variation σ. Each intensity point on the intensity versus time map is the intensity of a single pixel taken over several scans. **(C)** Equations used to calculate the number of particles (N) and brightness **(B)** as well as number of molecules (n) and intensity (*ε*). For derivation of these equations, see (Digman et al., 2008b). **(D)** Pixels from the image plotted as brightness versus intensity and the deduced aggregation state. Pixels in the red box are monomers, pixels in the green box are localized monomers, and pixels in blue are oligomers. **(E)** Shows the aggregation state plotted in its XY image to show the localization of aggregation. Images from Jackson et al., (2022a).

Number and Brightness (N&B) analysis is another fluorescence fluctuation spectroscopy technique that can be useful when measuring dimerization or aggregation (Digman et al., 2008b). N&B follows fluorescence fluctuations in the sample, similar to FCS. N&B and FCS differ in that FCS follows fluctuations at a single point while N&B follows fluctuations in a 2D image (Figure 10). Nolan and colleagues suggest that N&B will become a more popular method of analysis now that CRISPR-Cas9 is widely available to label endogenous proteins (Nolan et al., 2018). Our lab has used N&B to monitor stress granule formation in live cells which may not have been detected using less sensitive techniques such as confocal imaging (Qifti et al., 2022).

A third FFS method for studying protein-protein interactions is fluorescence cross-correlation spectroscopy (FCCS). In FCCS, the location of two fluorophores is simultaneously monitored so that the proximity of the two is measurable (Jameson et al., 2009). Ujlaky-nagy and János (2025) used FCCS to track the co-diffusion of the protein HER2. In the study, HER2 molecules were tagged with either AF488 or AF657 and their diffusion throughout the confocal volume was monitored. When a dimer or oligomer of AF488-HER2 and AF657-HER2 diffused through the confocal volume, their simultaneous movement could be monitored by plotting the count rate against time for both emission channels. Uncorrelated emission could also be tracked.: if AF488HER2 diffused alone, the count rate would not correlate with the count rate in the AF657 channel (Ujlaky-nagy and János, 2025). A benefit of FCCS over other fluorescence methods is that it can observe aggregation and diffusion over a longer period.

Förster Resonance Energy Transfer (FRET) is another fluorescence-based method for studying macromolecular interactions. FRET is a process in which a donor molecular transfers some of its energy to an acceptor molecule. For FRET to occur the chromophores must have overlapping excitation and emission spectra, be less than 10 nm apart, and properly oriented dipoles. Qualitative FRET can show the distribution of ion channels and conformational changes in a molecule, and quantitative FRET can measure the distance between the two molecules in the FRET pair (Wallrabe and Periasamy, 2005). Thus, when determining differentiation state, protein interactions can be identified using FRET. For example, Gromova and colleagues studied Smad protein interaction using FRET. In their experiments, cyan and yellow fluorescent proteins were fused with Smad1 and Smad4, and their interactions were imaged on a confocal microscope. The protein binding kinetics could be measured using this method, and the fluorescence lifetime (see below) were also measured (Gromova et al., 2007).

Fluorescence lifetime imaging microscopy (FLIM) is a modified FRET which has greater spatial and temporal accuracy. FLIM measures the average time a molecule stays in its excited state before returning to its ground state. Fluorescence lifetime is independent of the light path and the concentration of the fluorophore, making it a useful tool in live cells, where these variables are difficult to control. Our lab has used FLIM to study the differentiation state of PC12 cells (Garwain et al., 2020). CREB-calcium activity has also been studied using FLIM-FRET and FRET pairs of green and red CREB (Laviv et al., 2020).

**FIGURE 11:**
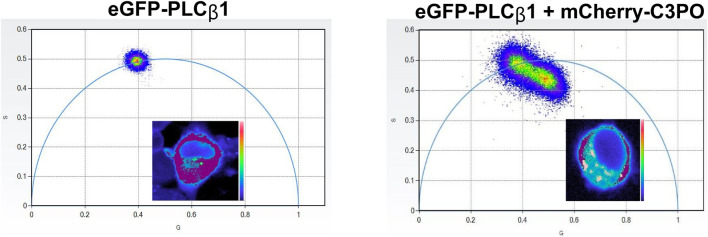
Images of an undifferentiated PC12 cells showing the lifetime distribution of PLCβ1 on phasor axes alone (left) and in the presence of a FRET acceptor with the corresponding cell images from (Qifti et al., 2021).

Phasor plots visualize FLIM-FRET through a two-dimensional histogram in which every pixel on an image is plotted on the phasor and every point on the phasor can be mapped onto the image (Digman et al., 2008a). This measurement requires that the sample be excited with phasemodulated light that allows lifetime determination by a reduction in fluorescence intensity and by a shift in the phase. These shifts measured directly from the detector can then be visualized in polar coordinates. For a single lifetime population, all points on the image will be seen as a single point on the phasor arc. Longer lifetime populations will lie closer to the Y-axis while shorter lifetime populations will lie further down the x-axis. If a FRET acceptor is present, then the donor population that participates in FRET will have a shorter lifetime, and the phasor plot will have a comet appearance (Figure 11) where the points inside the phasor arc will be the arithmetic mean of the two lifetimes (i.e., the donor alone and the donor fluorophore experiencing FRET). If the cellular localization of a particular point in the phasor is selected, then one can see where in the cell that FRET is occurring. The advantage of phasor plots is that the presence, degree and cellular distribution of FRET are assessed directly from the detector, thereby removing the complex calculations required including determining lifetime components.

Interactions between specific proteins can also be followed by biomolecular fluorescence complementation (BiFC) (Kerppola, 2006). In this method, one protein expresses GFP or an analog that is missing a portion of the molecular so that the barrel that houses the chromophore does not form. The protein partner is constructed to express the missing β strands so when the two proteins interact, the fluorescent GFP is generated giving off a fluorescent signal. The advantage of this method is that protein associations can be easily viewed by conventional fluorescence imaging. The disadvantages are that, like FRET, prior knowledge of the two proteins are required. Additionally, once the GFP reforms, it is kinetically trapped. Not only do the molecules remain together, but there is a potential for further associations. These sustained interactions may shift the outcome of the differentiation event.

## Summary

The incredible complexity driving cell differentiation and lineage selection is daunting, as we have describe for neuronal and fibroblast models. However, the potential ability to identify synergistic or antagonists processes has enormous potential for therapeutics. Here, we have described the ways that cell differentiation can be visualized in real time by monitoring shifts in protein localization and interactions associated with signaling pathways and by the appearance and disappearance of RNAs. Used with cell biological and computational techniques, these methods can be extended from cells to small organisms to ultimately understand differentiation.
